# Bibliometric analysis of research trends in agricultural soil organic carbon components from 2000 to 2023

**DOI:** 10.3389/fpls.2024.1457826

**Published:** 2024-12-04

**Authors:** Guolong Ge, Xuanyi Chen, Hexiao Ma, Xiangqian Zhang, Jingjing Shi, Xiaoxiang Wang, Xiaoqing Zhao, Manxiu Wang, Feng Xian, Zhanyuan Lu, Yuchen Cheng

**Affiliations:** ^1^ School of Life Science, Inner Mongolia University, Hohhot, China; ^2^ Inner Mongolia Academy of Agricultural and Animal Husbandry Sciences, Hohhot, China; ^3^ Key Laboratory of Black Soil Protection and Utilization (Hohhot), Ministry of Agriculture and Rural Affairs, Hohhot, China; ^4^ School of Life Science, Inner Mongolia University of Science and Technology Baotou Teachers University, Baotou, China

**Keywords:** soil organic carbon component, bibliometrics, visualization analysis, land-use pattern, CiteSpace

## Abstract

Soil organic carbon is a vital component of the soil carbon pool. Investigation of its composition and dynamics is crucial for enhancing carbon sequestration in soils and for stabilizing the global carbon cycle. In recent years, considerable research has focused on the interactions between soil organic carbon components and their responses to varied land use and agricultural practices. However, the mechanism of soil organic carbon sequestration and response characteristics of soil organic carbon components to soil carbon pools are still subject to some debate. To the best of our knowledge, no researchers have used bibliometric analyses to explore the field of soil organic carbon components. This study thus involved the use of bibliometric techniques to identify research hotspots in the study of organic carbon components over the last 23 years and future trends in research development. Specifically, we performed a comprehensive literature review of 607 documents pertaining to organic carbon components using the Web of Science database, covering the period from 2000 to 2023. Employing CiteSpace, we visualized and analyzed the data across national, institutional, disciplinary, and keyword dimensions. In this study, we conducted a comprehensive, systematic, and quantitative analysis of publications pertaining to organic carbon component research. The results indicate that researchers in the United States and China have substantially influenced the study of soil organic carbon components. Since 2000, the United States has pioneered the study of soil organic carbon components, establishing a foundational role in this field of research. Meanwhile, China leads with the largest number of publications and the most diverse research directions in this field. Among the institutions involved in such research, the University of Chinese Academy of Sciences has the highest number of publications. The investigation of soil organic carbon components within agricultural systems is inherently multidisciplinary, with the most comprehensive research being performed within the soil sciences discipline. At present, the focal areas of research on soil organic carbon components predominantly revolve around the impacts of straw return to fields, varying land-use changes, restoration of vegetation, and the reciprocal effects of soil organic carbon components on the restoration of vegetation. The findings of this work highlight the research hotspots within the field of soil organic carbon components and the emerging trends within this field. This work offers novel insights into the dynamics of soil organic carbon components, potentially guiding future studies in this vital field.

## 1 Introduction

Soil serves as a “carbon sink” in terrestrial ecosystems (Man et al., 2019). Research has demonstrated that terrestrial soils constitute the most substantial carbon repository on the Earth’s surface (Krahl et al., 2023). The estimated carbon content in soil is approximately 2,500 petagrams (Guo et al., 2020). The soil organic carbon pool represents a crucial component of the overall soil carbon reservoir (Zhao et al., 2021), with the organic carbon stored in soil constituting 60% of the global carbon reservoir (Rahmati et al., 2020). Moreover, the organic carbon reservoir within agroecosystems constitutes over 10% of the global terrestrial carbon stock (Hábová et al., 2019). Studies have revealed that the soil organic carbon pool in agroecosystems is the most important and dynamic reservoir within global terrestrial ecosystems (Dalal and Chan, 2001). Even minor fluctuations in this pool have profound impacts on the carbon cycle of ecosystems and the global climate (Amelung et al., 1997). Carbon sequestration in agricultural soils not only preserves soil productivity and enhances soil quality but also plays a crucial role as an ecological determinant in global biogeochemical cycles (Kirkby et al., 2014). It is thus particularly important to investigate soil organic carbon in agroecosystems to comprehensively understand carbon sequestration and stabilize soil carbon reservoirs.

Starting in the early 1970s, researchers began to investigate the composition and transformation processes of soil organic carbon reservoirs (Schlesinger, 1990). Researchers globally subsequently investigated the details of soil organic carbon components to elucidate the intricate interplay between soil organic carbon and various soil conditions (Easthouse et al., 1992). The organic carbon components within soil have become a particular focus within academia globally.

Bibliometrics offers an objective and dependable analytical framework for scholarly investigations (Zhou et al., 2024). By leveraging structured analysis of substantial datasets, bibliometrics enables researchers to draw inferences about temporal trends, delineate major research themes, identify shifts in the focus of research, and determine which research institutions are leading particular fields (Yin et al., 2020). Given these advantages, bibliometrics is used here to analyze soil organic carbon components in agricultural systems. Bibliometric analysis constitutes a sophisticated method of quantitative research, offering meticulous examination and insightful analysis of research outcomes within specialized domains (Yang et al., 2023). The primary objective of literature analysis is to acquire comprehensive insights into various dimensions such as countries, institutions, keywords, and disciplines (Ferreira et al., 2016). This information facilitates subsequent performance evaluations and analyses, serving as a cornerstone for assessing the evolution of research within a given field (Ouyang et al., 2018). Bibliometrics represents a rigorous quantitative methodology for analyzing publications through the application of mathematical statistics (Li et al., 2021).

Since the advent of the 21st century, research on soil organic carbon components has deepened and broadened in its scope. Between 2000 and 2008, researchers both within China and internationally undertook extensive and comprehensive investigations into the correlations between soil organic carbon components and their responses to diverse land-use typologies, including land cover and land management practices. Investigations ranged from analyses of the types and concentrations of soil organic carbon components across various parts of the world to assessments of the impacts of changes in farming systems and nutrient management strategies on the composition of soil organic carbon in lands used for agriculture. As global ecosystems undergo transformations, the focal points and directions of research have accordingly evolved. In recent years, there has been a significant surge in studies investigating the intersection of soil organic carbon components and their responses to varied land-use patterns, including the effects of deforestation on forest soil organic carbon, the impacts of grassland degradation, and the outcomes of efforts to restore vegetation on organic carbon levels.

The purpose of this study is to perform a comprehensive and systematic bibliometric analysis of soil organic carbon components and to address the knowledge gaps in the bibliometric reviews performed on this topic to date. Specifically, the main objectives of this study are as follows: 1) to clarify the trajectory of research into soil organic carbon components spanning from 2000 to 2023 against the backdrop of globalizing tendencies within academic research, 2) to consider the main research themes within this field and their distinguishing features, and 3) to highlight the research avenues that now appear to be particularly valuable, based on an analysis of nascent trends.

## 2 Materials and methods

### 2.1 Data sources

The Web of Science is a major multidisciplinary core journal database, which is an invaluable resource for academic research across various fields (Martín-Martín et al., 2018). In this work, two databases in the core collection of the Web of Science were selected as data sources, namely, the Science Citation Index Expanded (SCIE) and Social Sciences Citation Index (SSCI) within the Web of Science Core Collection. To retrieve relevant reports, the following search formula was used: TS = [(“organic carbon fractions” OR “organic carbon component” OR “organic carbon and its fractions”) AND (“soil*” OR “land*” OR “farmland*” OR “cropland*” OR “cultivated land*”)]. The search (performed on November 6, 2023) included manuscripts published from January 1, 2000, to November 6, 2023. This search retrieved 626 documents, comprising 613 articles, eight reviews, and three conference abstracts, among others. A meticulous screening process was then performed to select only research articles and those written in English (Yao et al., 2020), which yielded 607 articles for further analysis.

### 2.2 Research methodology

The retrieved articles were exported in plain text format along with complete records and references and subsequently imported into CiteSpace 6.1.R6, a Java-based application, for further bibliometric and visual analyses (Kushwah et al., 2019). CiteSpace is a sophisticated visualization instrument that can determine academic architecture and emerging trends across domains of knowledge, quantitative research methodologies, and intelligence (Zhong et al., 2020). Knowledge graph visualization was performed following the main procedural steps of CiteSpace, which include time slicing, thresholding, modeling, pruning, merging, and mapping. Core concepts of CiteSpace encompass burst detection, centrality, and heterogeneous networks, aiding rapid visualization of the state of research, hotspots, and new research frontiers (Damar et al., 2018). The nodes included in the various maps represent authors, institutions, countries, or keywords. The size of these nodes represents the frequency of occurrence or citation, while their color indicates the year of occurrence or citation (Şahin and Yılmaz, 2022). Additionally, purple bordering of nodes is used to signify research hotspots or turning points within a field (Donthu et al., 2021). Centrality serves as a fundamental metric for analyzing the significance of keywords (Zhang et al., 2011). A node is considered to be “central” if its centrality exceeds 0.1, indicating that it is particularly important and influential in the field (Sabe et al., 2022). Meanwhile, a keyword acting as a mediator between articles A and B holds a pivotal position or serves as a connection among multiple articles, playing a crucial role (Chen, 2006). Nodes with mediator centrality exceeding 0.1 are designated as key nodes (Pan et al., 2018). CiteSpace can also address connections or working relationships between papers, aiding users in bridging cognitive gaps and identifying crucial points and future trends in the research field (Liao et al., 2018). Thus, CiteSpace-based bibliometrics was employed here to analyze papers in the literature pertaining to soil organic carbon components. Key manuscripts were also read critically to facilitate a thorough analysis of pivotal studies and to offer insightful perspectives on the topic.

### 2.3 Data analysis

Employing the function for analyzing the results of the Web of Science search, the retrieved literature was categorized and archived. Microsoft Excel was subsequently used to count annual publications, delineating the countries, researchers, and institutions from which publications were derived. CiteSpace (version 6.1.R6) was also used to extract important noun phrases from the titles, abstracts, and keywords of the literature. This facilitated an analysis of common and emerging words within the literature. Subsequently, the trajectory of the research field of soil organic carbon components and the focal points within it were determined by drawing plots using the aforementioned software tools. Within CiteSpace, a period of 23 years was designated, with each time node representing a single year. Node types encompassed Country and Keyword, among others, with node strength defaulted to Cosine. The Minimum Spanning Tree was chosen as the network cropping functional area for graphical analysis. Moreover, when analyzing changes in the keywords used over time, the time zone was selected.

## 3 Results and discussion

### 3.1 Analysis of publication trends

**Figure 1:**
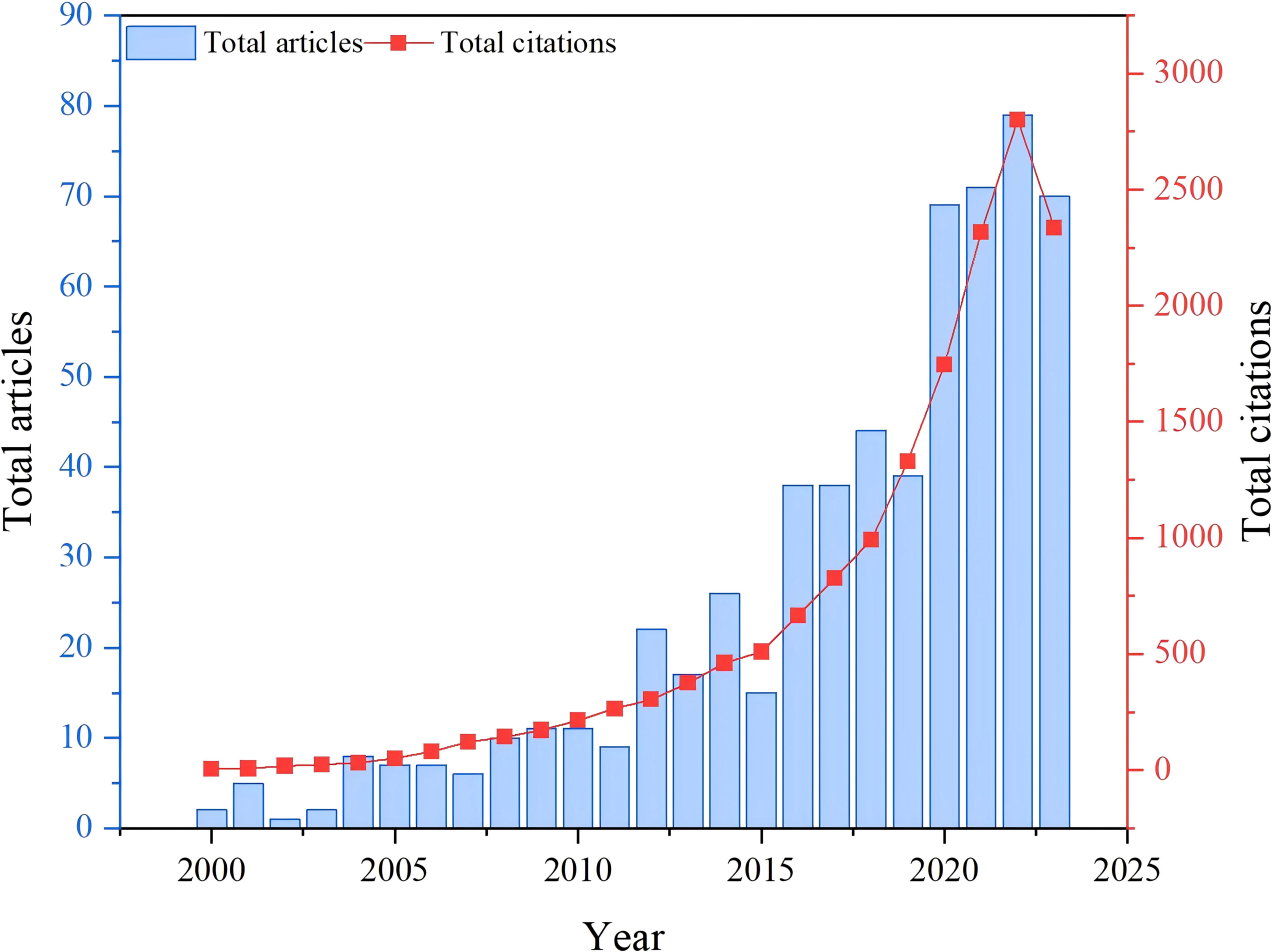
Time course of the total number of publications and citations in the Web of Science database.

The trajectory of the amount of published literature within a particular discipline provides a valuable gauge of advances in that research field (Thelwall, 2008). Plotting the number of published documents over time is thus useful for evaluating the research landscape within a particular field, helping to interpret and predict dynamics and trends (Cheng et al., 2022). **Figure 1** depicts the annual distribution of published literature describing research on soil organic carbon components within the Web of Science over the last 23 years. It reveals that the average number of publications on this subject annually during this period was only 26. Overall, research concerning soil organic carbon components has notably grown and is progressing steadily. Notably, in recent years, the numbers of both publications and citations about soil organic carbon components have steadily grown. Prior to 2011, the literature output was relatively sparse, hovering around a modest seven articles annually. However, after 2018, there was a notable surge, with approximately 62 articles being published per year on average. In line with this, the number of citations increased markedly from only four to 2,337 during this period. Although the number of studies is still quite limited compared with that in other fields, the research on soil organic carbon components has obtained many high-quality results. Concurrently, based on the research trajectory, it is anticipated that ample research opportunities will occur in this field in the coming years, ensuring continued growth in the exploration of soil organic carbon components.

### 3.2 Country analysis

**Figure 2:**
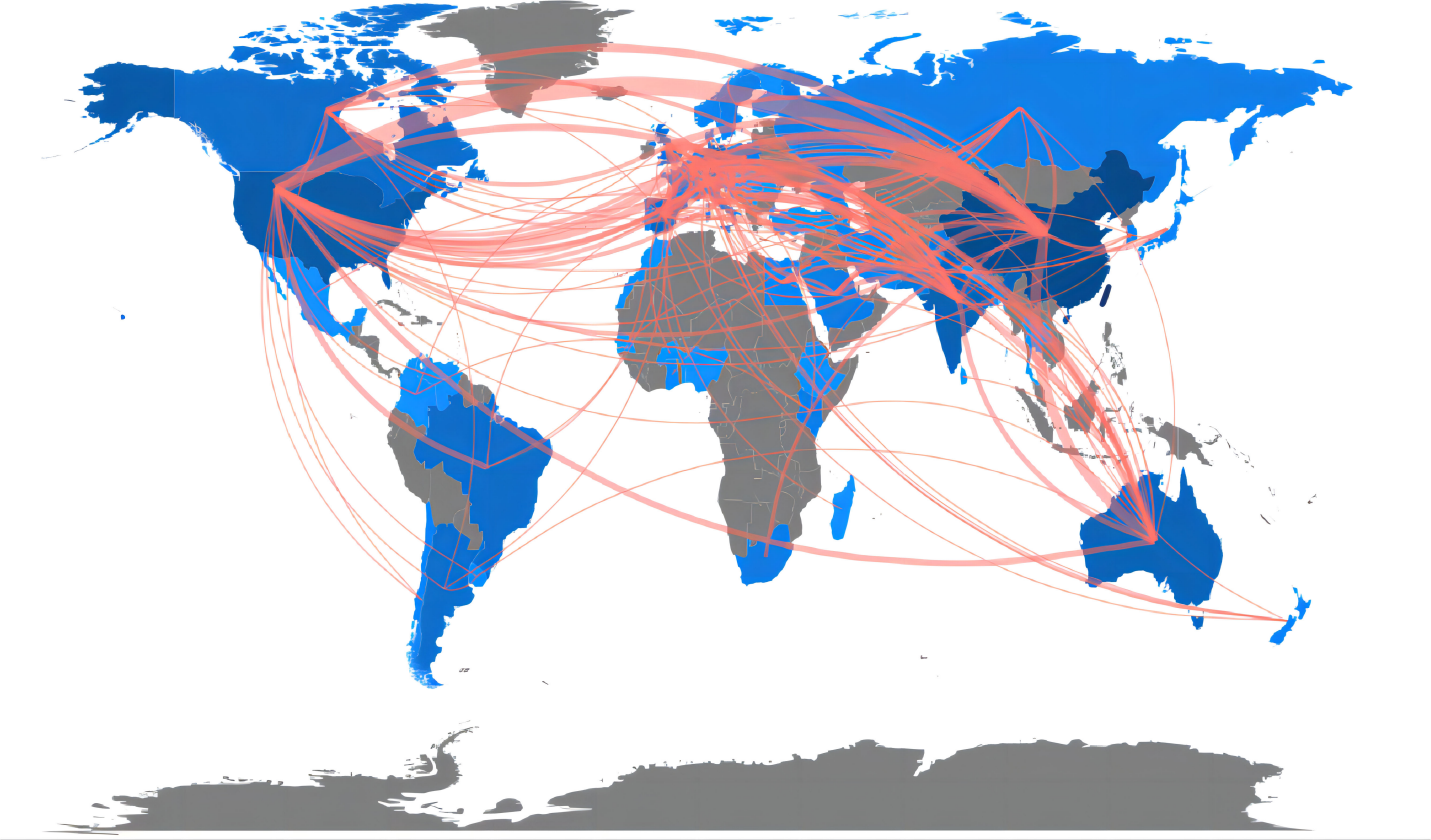
Global collaboration network.

**Figure 3:**
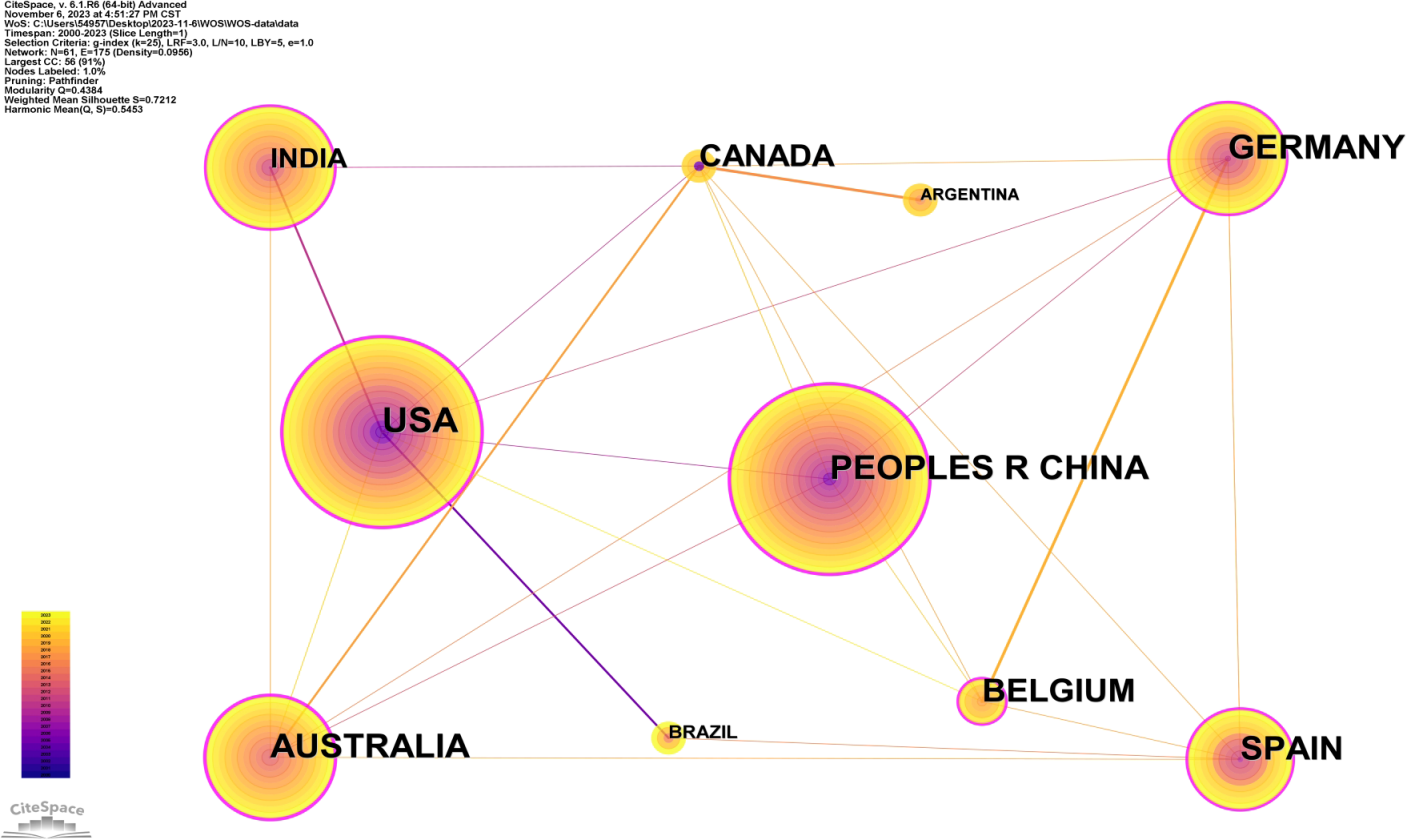
Collaboration network map of countries.

By conducting analyses of the countries where research within the field is performed, we not only can discern the countries that are most important within the field of research on soil organic carbon components but also can illuminate the interactions between researchers and departments and collaborations among countries (**Figure 2**). Consequently, here, we chose to perform an analysis of countries associated with such research using CiteSpace as the analytical framework. We designated the Time Slicing parameter spanning the period 2000–2023, with Years Per Slice set at 1. This yielded 61 network nodes and 175 connecting lines, resulting in a density of 0.0956 for the country map analysis (**Figure 3**). The size of the purple ring signifies the degree of centrality, a metric evaluating the significance of a node’s placement in the network (Narin, 1994).

**Table 1** displays the top 10 countries ranked by frequency of publications. Here, “frequency” denotes the number of publications derived from each country, while “centrality” delineates the country’s prominence within the field. Centrality clearly serves as a descriptor of a country’s connectivity: the greater a country’s connections, the higher its centrality, indicating a more robust research profile and a more influential position within the field.

**Table 1:** Top 10 countries by the number of publications.

Count	Centrality	First publication year	Country
367	0.38	2003	PR China
86	0.38	2000	USA
50	0.13	2007	India
38	0.32	2001	Australia
34	0.15	2003	Germany
31	0.19	2006	Spain
16	0.01	2005	Brazil
16	0.1	2001	Canada
15	0	2001	Argentina
11	0.12	2015	Belgium

Examination of the frequencies in **Table 1** reveals that the People’s Republic of China (PRC) stands out as the country with the largest number of articles in this field (n = 367), far surpassing the numbers from other countries. This underscores the PRC’s significant dedication to research on soil organic carbon components and the country’s rapid advance in this field. The PRC’s centrality measure is 0.38, also implying that Chinese researchers and institutions relatively frequently undertake international collaborations. The United States ranks second in publication volume (n = 86), which was the leading nation in research on organic carbon components in the 20th century, having a centrality value of 0.38. This underscores the extensive research and international collaborations of American researchers and institutions in this field. Notably, India’s centrality, despite its ranking of 3 in terms of publication volume, differs significantly from that of the United States and PRC, suggesting that Indian researchers and institutions are less involved in international collaborations. Meanwhile, Brazil and Argentina were found to have minimal international collaboration and low centrality. Conversely, Australia, Germany, Canada, Belgium, and Spain achieved centrality levels above 0.1, suggesting that multinational researchers are actively involved in their research endeavors. The leading three nations in terms of publication volume initiated their research in this field in 2000, 2001, and 2007. It is noteworthy that India, despite having a later start, has made significant contributions to this field of research, suggesting the substantial potential and rapid development of Indian researchers in this field.

### 3.3 Institutional analysis

**Figure 4:**
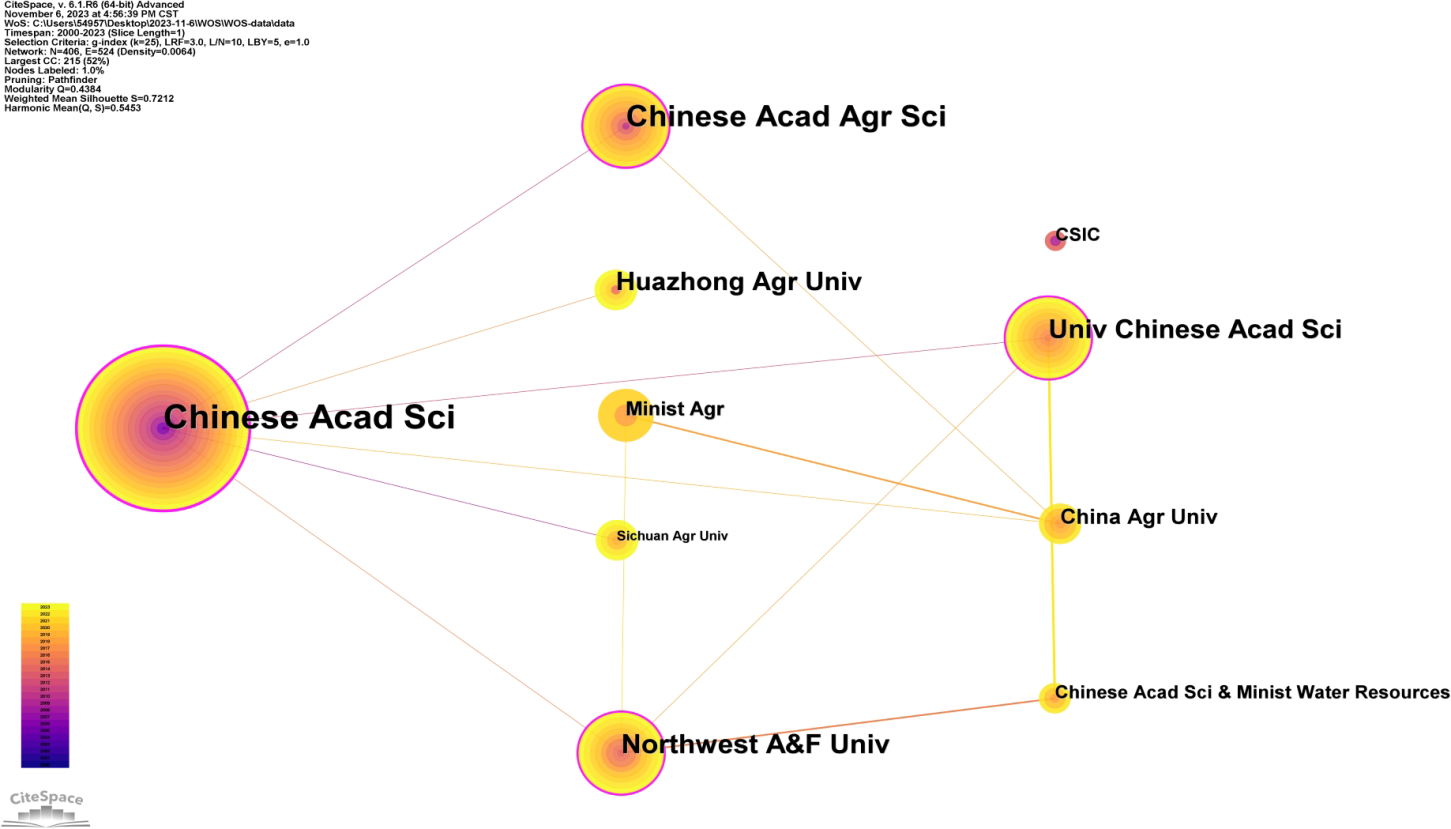
Collaboration network map of institutions.

To analyze the institutions involved in research on soil organic carbon components using CiteSpace, we assembled 406 network nodes and 524 connecting lines, resulting in a density of 0.0064 for the institutional analysis map (**Figure 4**). As shown in **Figure 4**, the nodes in the graph are quite dense, and there are much data, with a total of 524 connections. This suggests a comparatively high frequency of institutional cooperation within the field of soil organic carbon components, with most research institutions engaged in collaborative efforts. Furthermore, there are some distinct geographical patterns in the collaborations, underscoring the importance of ongoing cross-institutional research and cooperation to facilitate academic exchange within the field of soil organic carbon components. This study presents the top 10 institutions in the field of soil organic carbon components based on article count. According to **Table 2**, the Chinese Academy of Sciences leads with 107 articles and a centrality of 0.31, surpassing other institutions. Conversely, the centrality of Consejo Superior de Investigaciones Científicas (CSIC) is 0.00, indicating that it is less prominent in the network. Huazhong University of Science & Technology demonstrates high article frequency but limited cooperation with other institutions. Notably, the earlier publication history of the Chinese Academy of Sciences reflects its significant and longstanding contributions to the field of soil organic carbon components. Meanwhile, Northwest A&F University secures second place with a frequency of 39 articles and a centrality of 0.15, despite entering the field of soil organic carbon components at a relatively late stage. This suggests recent in-depth research in this field at Northwest A&F University, guiding the current direction of cutting-edge research.

**Table 2:** Top 10 institutions by the number of publications.

Count	Centrality	First publication year	Institution
107	0.31	2004	Chinese Acad. Sci.
39	0.15	2014	Northwest A&F Univ.
38	0.11	2012	Univ. Chinese Acad. Sci.
38	0.1	2010	Chinese Acad. Agr. Sci.
16	0.07	2011	Huazhong Agr. Univ.
13	0.01	2009	Sichuan Agr. Univ.
12	0.03	2012	China Agr. Univ.
10	0.01	2016	Chinese Acad. Sci. & Minist. Water Resources
10	0.01	2018	Minist. Agr.
9	0	2008	CSIC

### 3.4 Analysis of academic disciplines

**Figure 5:**
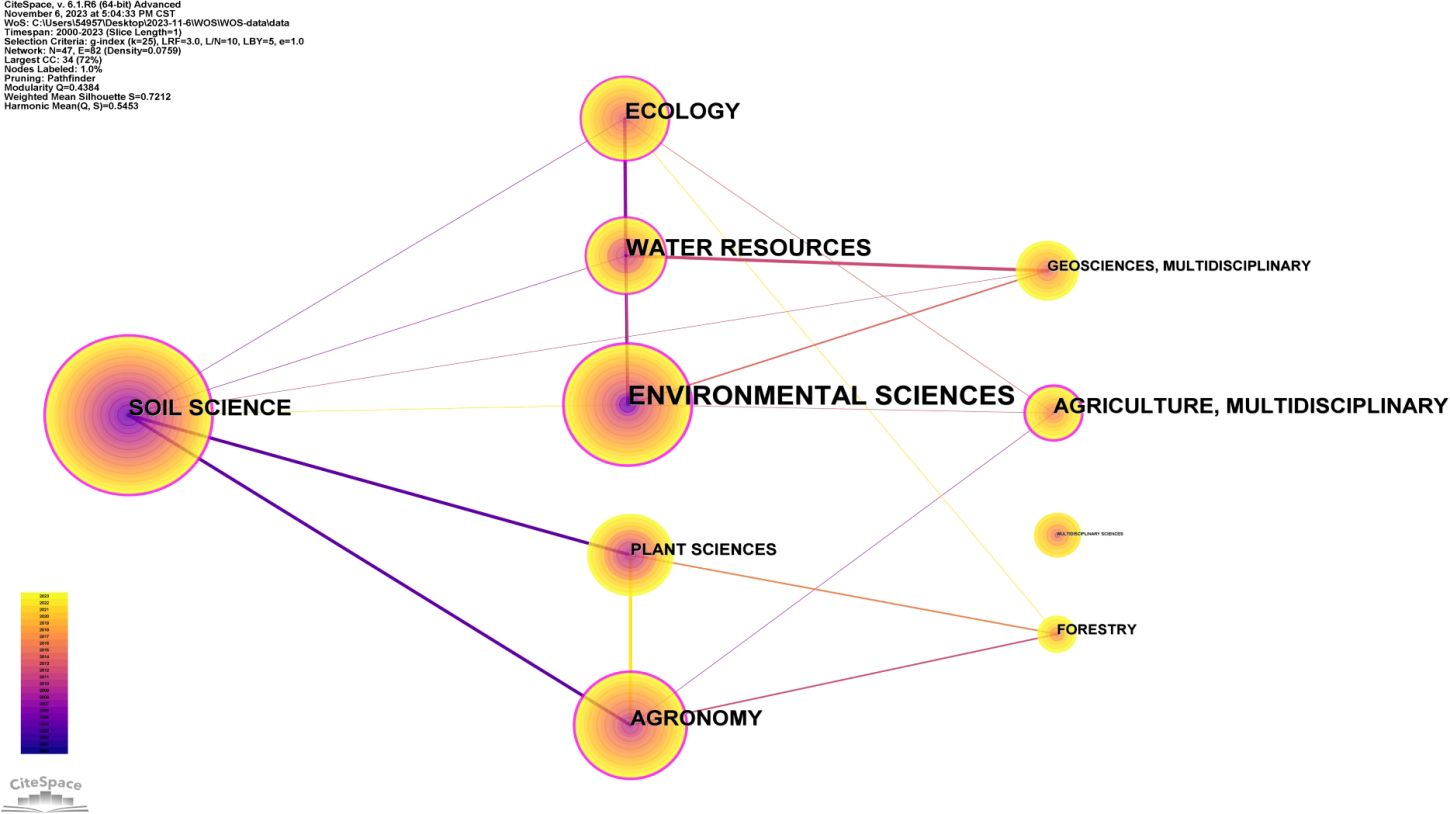
Collaboration network map of academic disciplines.

By performing an analysis of academic disciplines in CiteSpace, we identified 47 network nodes and 82 connecting lines, resulting in a density of 0.0759 in the disciplinary analysis map (**Figure 5**). In this study, we categorized the top 10 academic disciplines involved in research on soil organic carbon components. **Table 3** reveals that such research has primarily been performed within disciplines such as soil science, environmental science, agricultural science, and plant science. Soil science tops the list with 302 articles published since 2000, representing 34.1% of the total, and centrality of 0.30. Environmental science follows, with 201 articles published since 2000, accounting for 22.2% of the total, and centrality of 0.36, surpassing that of soil science. This suggests that, while environmental science researchers have published fewer articles than those working in soil science, the interdisciplinary connections in the former field are stronger, indicating growing interest across various fields in soil organic carbon components.

**Table 3:** Top 10 academic disciplines by the number of publications.

Count	Centrality	First publication year	Academic disciplines
308	0.3	2000	Soil science
201	0.36	2000	Environmental sciences
90	0.24	2003	Agronomy
77	0.01	2003	Plant sciences
49	0.14	2003	Water resources
45	0.16	2007	Ecology
41	0	2003	Geosciences, multidisciplinary
35	0.27	2008	Agriculture, multidisciplinary
31	0.01	2011	Forestry
26	0	2005	Multidisciplinary sciences

### 3.5 Analysis of research hotspots

**Figure 6:**
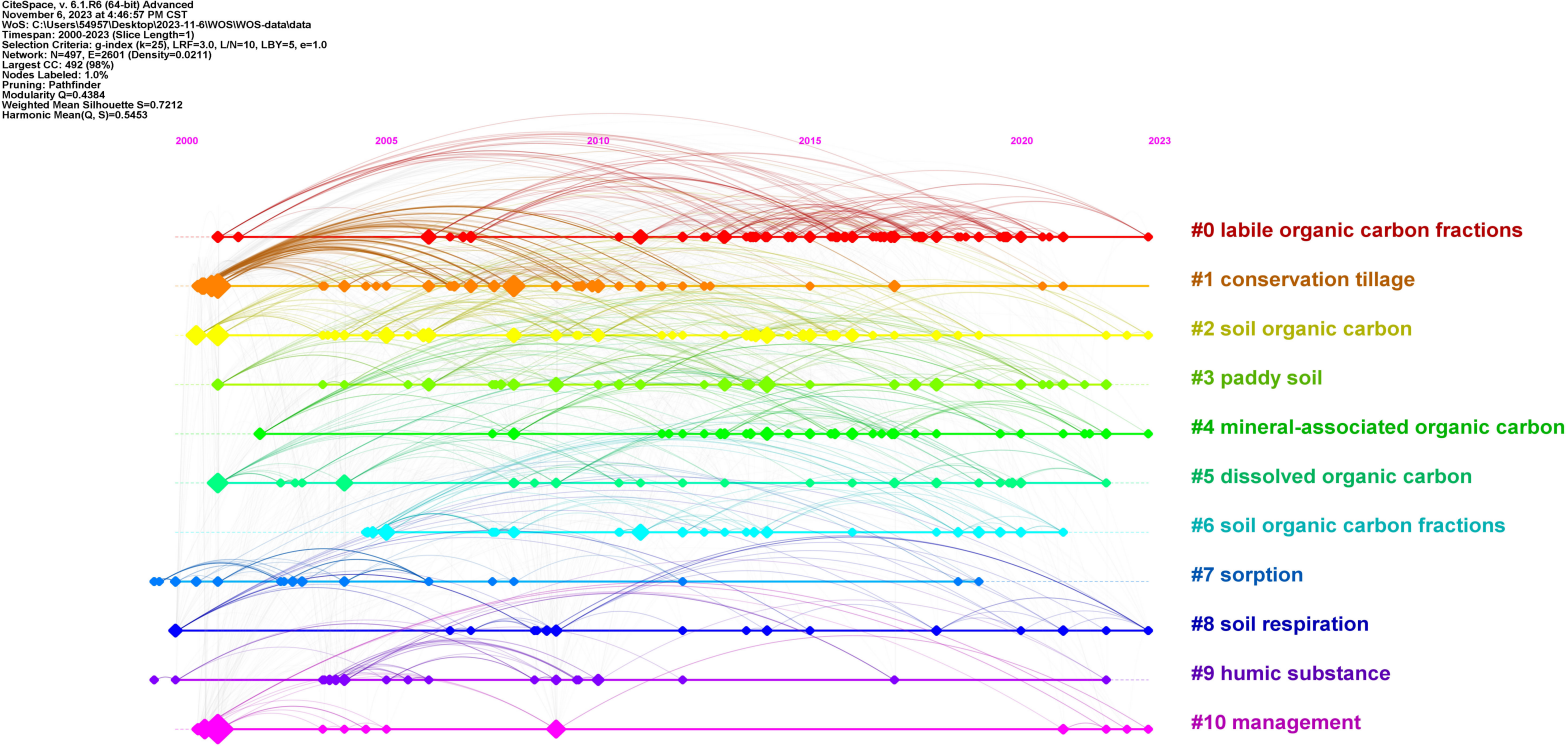
Keyword clustering map for soil organic carbon components.

Literature keywords represent pivotal terms extracted from articles that succinctly encapsulate their themes. Keyword clustering can thus be applied to elucidate the principal trajectory of a research field and its prominent themes (Zhou et al., 2023), thereby facilitating analysis and visualization of research hotspots pertaining to “soil organic carbon components”. Here, keywords are clustered and analyzed using CiteSpace. In this study, the keywords were clustered and analyzed using CiteSpace, the cluster option was selected, and the pathfinder algorithm was used to crop the connecting lines in order to ensure that the clusters were classified reasonably. The results are shown in **Figure 6**, which reflects the research themes in the field of organic carbon fractions in the last 23 years. This study identified the following 11 research themes: #0 labile organic carbon fractions, #1 conservation tillage, #2 soil organic carbon, #3 paddy soil, #4 mineral-associated organic carbon, #5 dissolved organic carbon, #6 soil organic carbon fractions, #7 sorption, #8 soil respiration, #9 humic substance, and #10 management. The different clusters were associated with distinct keywords.

The focal research themes (#0 labile organic carbon fractions, #5 dissolved organic carbon, #6 soil organic carbon fractions, and #10 management) centered on elucidating the “Response mechanisms of soil organic carbon fractions to diverse land management practices”. The investigations concentrated on assessing the impacts of agricultural management practices, including tillage practices, crop rotation, blending organic and inorganic fertilizers, and irrigation management, on soil organic carbon fractions. Among the articles analyzed in this study, we found one on a study of paddy soils in southwest China, in which Shao et al. discovered that judicious land management practices had the potential to enhance the sequestration of organic carbon components, particularly in the surface soil layers. Specifically, over a period of 15 years, practices such as no-tillage, ridge cropping, and rice–wheat rotation led to substantial enhancements in the levels of easily oxidizable organic carbon, dissolved organic carbon, particulate organic carbon, and microbial carbon within the soil layer at a depth of 0–20 cm, with the most pronounced increases observed at 0–10 cm. At a depth of 0–10 cm, conservation tillage-wheat exhibited higher levels of soil oxidizable organic carbon, dissolved organic carbon, particulate organic carbon, and microbial carbon compared with traditional tillage-fallow, traditional tillage-wheat, and conservation tillage-fallow (Shao et al., 2009). We collated the data and conclusions of the publications. This was mainly attributed to the improvement of soil microenvironment (soil structure, moisture, porosity, and temperature) by no-tillage and ridging, which increased dissolved organic carbon and particulate organic carbon (Gao et al., 2008). The changes in the soil microenvironment further increased soil microbial and enzyme activities, resulting in an increase in easily oxidizable organic carbon and microbial biomass carbon (Zou et al., 2024). Rice–wheat rotation ensures the continuity of vegetation cover and increases particulate organic carbon due to the return of stubble and roots to the field. This rotation pattern also leads to the accumulation of more dead plants, roots, and low-molecular-weight root exudates, which significantly increases soil microbial populations and thus elevates the microbial biomass carbon content (Wei et al., 2022). No-tillage, monopoly, and rice–wheat rotations improved soil water-stability aggregates by 20%–45%, and the improvement of soil aggregates protected soluble soil organic matter from microbial attack, resulting in an increase in dissolved organic carbon (Huang et al., 2023). Conservation tillage-wheat conditions were associated with higher levels of all organic carbon fractions than in the other treatments, which was mainly attributed to the frequent cycles of flooding and drainage of the rice soil upon alternating between the rice- and non-rice-growing periods, and the soil moisture at the top of the ridge was near field capacity during the non-rice-growing period. Under these conditions, there are increases of surface soil temperature, plant residue, and root matter (Yan et al., 2022). These findings suggest that no-tillage, ridge cropping, and the integrated cultivation of rice and wheat represent optimal land management strategies for rice production, leading to paddy soils characterized by elevated levels of easily oxidizable organic carbon, dissolved organic carbon, particulate organic carbon, and microbial biomass carbon sequestration. This work helps us to understand the effects of nutrient management strategies on soil organic carbon storage and unstable organic carbon components and to improve soil carbon sequestration and mitigate climate change. Moreover, following 26 years of the field application of organic fertilizer and mineral composite fertilizer in the North China Plain, Li et al. revealed a significant reduction in soil bulk density. Their findings showed that the organic carbon reserves, active organic carbon components, mineral fertilizers, and soil organic carbon total amount increased (Li et al., 2018).

The thematic focus (#1 conservation tillage, #2 soil organic carbon, and #3 paddy soil) centers on elucidating the “mechanisms governing soil health and functionality in reaction to conservation tillage practices”. The study focused on how to adjust conservation tillage to maximize the positive response of soil organic carbon based on the characteristics of the local environment where the researchers were located. According to a report on threats to the world’s soils, it is predicted that approximately a quarter of the world’s land will undergo degradation. The threats to soil should be a particular concern in water-scarce areas, as this soil is crucial for food and economic security in countries around the world (Montanarella et al., 2016). In another study, Zhu et al. discovered that adopting no-tillage techniques as part of conservation tillage markedly increased the organic carbon reserves within the tillage layer, while the incorporation of straw residues notably increased labile organic carbon fractions and particulate organic carbon components (Zhu et al., 2022). The studies reported in many of the articles analyzed here have shown that conservation tillage can improve soil structure, increase soil organic carbon storage, and improve the health of the soil (Bohoussou et al., 2022).

The themes (#4 mineral-associated organic carbon and #7 sorption) focus on the “dynamics of mineral-associated organic carbon components under climate and land-use changes”. It has been revealed that there is a close relationship between soil mineral-associated organic carbon, particulate organic carbon, and aggregate, which in turn influences the dynamic changes in soil organic carbon (Zhang et al., 2023b). Among the numerous studies included in the analysis described here, one investigated the dynamics and enrichment of organic carbon components in sediments influenced by varying rainfall patterns and slope lengths during periods of climate change, alongside their interactions with soil erosion and grain size distribution. It was shown that the concentrations of particulate organic carbon and mineral-associated organic carbon within the soil organic carbon composition remained relatively consistent under different rainfall patterns and slope conditions, with mineral-associated organic carbon being significantly enriched under conditions of moderate rainfall (Li et al., 2023). The primary reason for this is the decrease in mineral-associated organic carbon with increasing slope lengths. Additionally, rainfall and slope length are key parameters that directly impact the process of soil erosion and indirectly affect the distribution of sediment grain size. This in turn leads to changes in the content of organic carbon components and their loss, with mineral-associated organic carbon being the component that is particularly prominently lost (Jiang et al., 2019). Wang et al. demonstrated that land-use transitions contribute to the decomposition of certain recalcitrant organic carbon components, resulting in increased levels of such components, including mineral-associated organic carbon, within the mineral soil profile after tillage activities. Moreover, recalcitrant organic carbon components, including mineral-associated organic carbon, exhibit prolonged longevity in storage when subjected to land management practices involving keeping the land fallow (Wang et al., 2017).

The thematic emphasis (#8 soil respiration) centers on elucidating “soil respiration dynamics in response to diverse land-use alterations across various geographical regions”. Elevations in soil respiratory components, encompassing heterotrophic and autotrophic respiration, are linked to variations in soil organic carbon, which are primarily driven by increases in the abundance of microbes and α-diversity, along with shifts in microbial β-diversity that amplify the organic carbon components and respiratory components associated with changes in land use (Ren et al., 2018). Among the literature included in this analysis, we identified a study examining land-use changes and utilization in subtropical forest regions. Specifically, in their investigation of the impacts of heightened carbon dioxide levels and nitrogen supplementation on soil organic carbon components within subtropical forests, Chen et al. demonstrated that, even within nitrogen-enriched subtropical forest ecosystems, there is a need for nitrogen supplementation for the accrual of carbon in soil under elevated CO_2_ conditions (Chen et al., 2012).

The theme (#9 humic substance) centers on the “variations in soil humus attributable to diverse farming practices and land-use modifications”. Among the many articles on this theme included in the current study, we found one study on the changes in soil organic carbon components following the application of organic materials (OMs) in semi-arid soils under drip irrigation and plastic mulch conditions. Two years of field experiments showed that, after the continuous application of OMs, the contents of labile organic carbon and recalcitrant organic carbon increased by 3.2%–8.6% and 5.0%–9.4%, respectively, compared with the levels at baseline (Hu et al., 2018). This was mainly attributable to lignin, polyphenol, total water-soluble organic matter, water-soluble humic acid, humic-like substance, and humic acid-like contents among the initial OMs, playing important roles in soil organic carbon and recalcitrant organic carbon. Meanwhile, in another study of humus changes caused by the reclamation of grassland for farmland, it was found that the quality of soil humus decreased. This was primarily ascribed to the declining ratios of the optical densities, or absorbances, of humic and fulvic acid solutions at respectively 465 and 665 nm (E4/E6 ratios), coupled with the hue coefficient (Δlog K values), which reflects the logarithmic disparity in absorbance between 400 and 600 nm for the humic (fulvic) acids in the solution (Wu et al., 2022a). This study deepens our knowledge of the effect of grassland reclamation on soil organic carbon and its fraction in alpine-cold soils at high altitudes, having major implications for the long-term maintenance of the global carbon balance.

### 3.6 Analysis of research frontiers and trends

**Figure 7:**
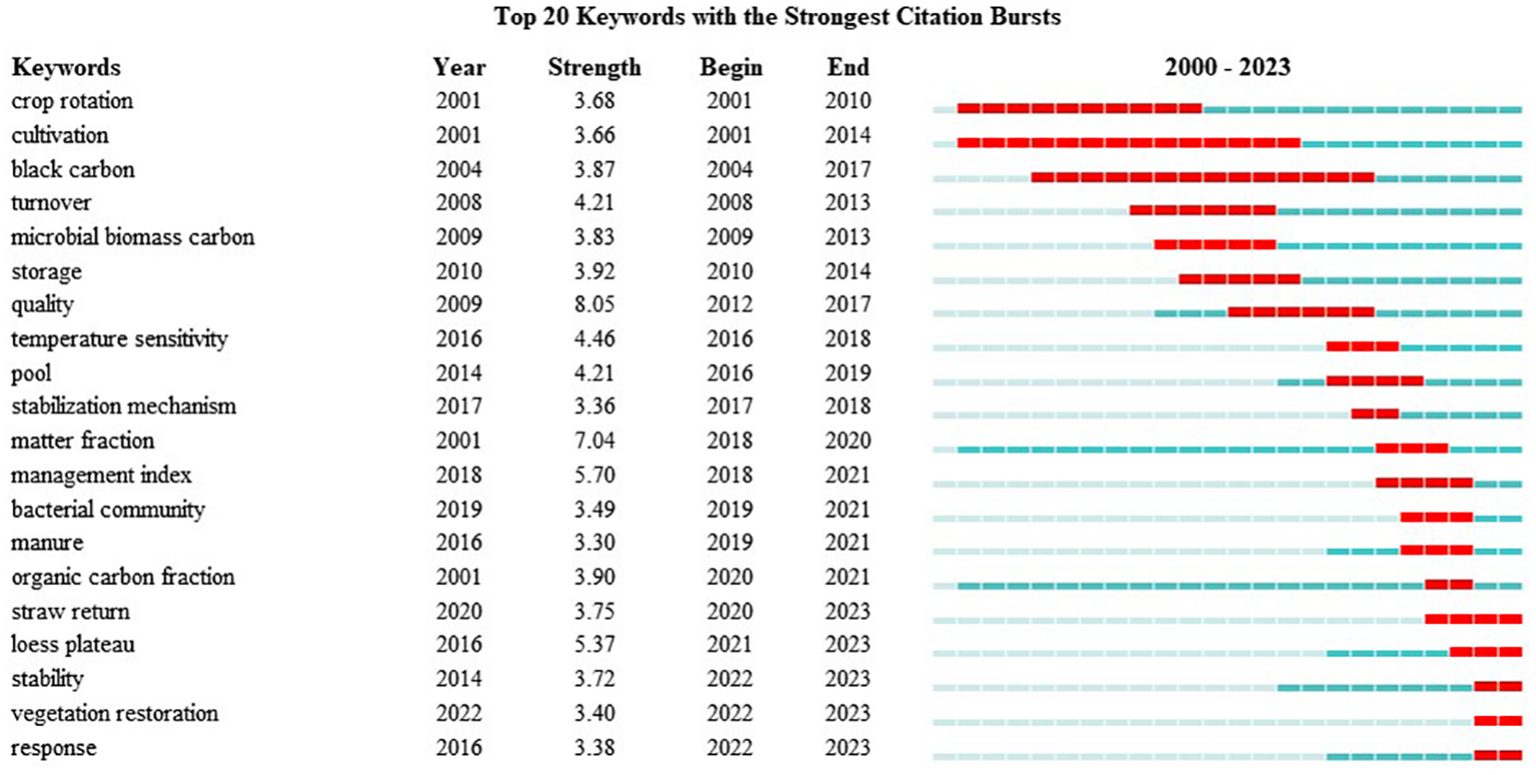
Mapping of 20 keywords with the most intense citation bursts.

This study utilized the Bursts detection algorithm of the CiteSpace software to map keyword hotspot evolution, delineating the emergence of keywords within the field of organic carbon component research in the Web of Science, as depicted in **Figure 7**. This study generated the top 20 keywords pertaining to organic carbon components, based on their emergence intensity, and the specific emergence intensities along with hotspot durations are presented in the figure. A blue line signifies the time interval of emergence, whereas a red line denotes the time period during which a burst was detected within a specific topic category. The years of commencement and conclusion of the burst can thus clearly be distinguished (Fu et al., 2022).

**Figure 7** illustrates the hotspot period when the field of soil organic carbon components was particularly active from 2001 to 2019. Throughout this period, the primary research focuses included crop rotation, cultivation, black carbon, turnover, microbial biomass carbon, storage, quality, pool, and stabilization mechanisms. Meanwhile, following this period, 2020 marks the emergence of new topics and hotspots, encompassing organic carbon components, straw return, the Loess Plateau, stability, restoration of vegetation, and response.

Keyword analysis revealed that contemporary researchers are increasingly directing their attention to the impact of straw return on soil organic components and the mechanisms underlying the response of soil organic carbon components to vegetation restoration. Specifically, in this study, straw return, microbial biomass carbon, and manure emerged as central areas of investigation. For example, in a short-term straw return study spanning 3 years conducted in 2016, Chen et al. discovered that the primary factors influencing the structure of the soil microbial community are microbial biomass carbon and dissolved organic carbon. The study’s findings indicated that, in the context of short-term straw return, soluble organic carbon and microbial biomass carbon emerged as the most sensitive indicators for assessing changes in soil organic carbon within a rice–wheat two-maturing system (Chen et al., 2017). Meanwhile, in a comprehensive long-term study spanning 30 years conducted in 2015, Zhao et al. discovered that high levels of straw return (9,000 kg ha^−1^) led to significant increases in both light and heavy component organic carbon concentrations (Zhao et al., 2016). Moreover, in a global stoichiometric study of straw return to maintain soil ecology, Liu et al. found that the return of crop residue to cropland soils mitigated carbon limitation caused by inorganic fertilizer inputs by increasing soil carbon content, mitigated soil organic matter mineralization, and augmented soil carbon, nitrogen, and phosphorus levels, thus contributing to ecological protection and climate change mitigation (Liu et al., 2023). With the ongoing expansion of the body of theoretical research pertaining to organic carbon components, vegetation restoration, response, and related topics, these issues have drawn widespread attention. Notably, research hotspots in this field have emerged within China’s tropical karst, subtropical karst, and the Loess Plateau in north-central China. For example, in their investigation of China’s karst region, Zhang et al. examined the response of soil organic carbon components to the restoration of natural vegetation in tropical karst regions. Their findings revealed significant correlations between the stage of vegetation restoration and soil organic carbon and its components: specifically, as the restoration progressed, soil organic carbon content gradually increased (Zhang et al., 2023a). Moreover, within the subtropical karst region, Wu et al. identified positive correlations between soil organic carbon components and soil biomass, iron content, and globularin-related soil protein. Additionally, they highlighted that soil physicochemical and microbial interaction processes significantly influence the persistence of soil organic carbon components. While various soil carbon indexes can be used to evaluate vegetation recovery, this study’s findings highlighted permanganate oxidizable carbon as the most sensitive index during the change of the soil organic carbon component (Wu et al., 2022b). Meanwhile, in a 2022 study on the semi-arid Loess Plateau in north-central China, Ghani et al. found that the continuous supply of organic matter after returning farmland to forest changed the distribution of soil organic carbon among different components, and the balance between the input and decomposition of organic matter increased the total amount of soil organic carbon, which promoted the accumulation of soil organic carbon in different vegetation types (Ghani et al., 2023). The findings of this study suggest that the long-term restoration of natural vegetation is pivotal for the sequestration and cycling of soil organic carbon in the Loess Plateau region of China. The data acquired by researchers promote the evaluation of vegetation restoration and soil carbon accumulation across varying scales, offering an effective approach for enhancing carbon sequestration and soil conservation.

## 4 Conclusion

**Figure 8:**
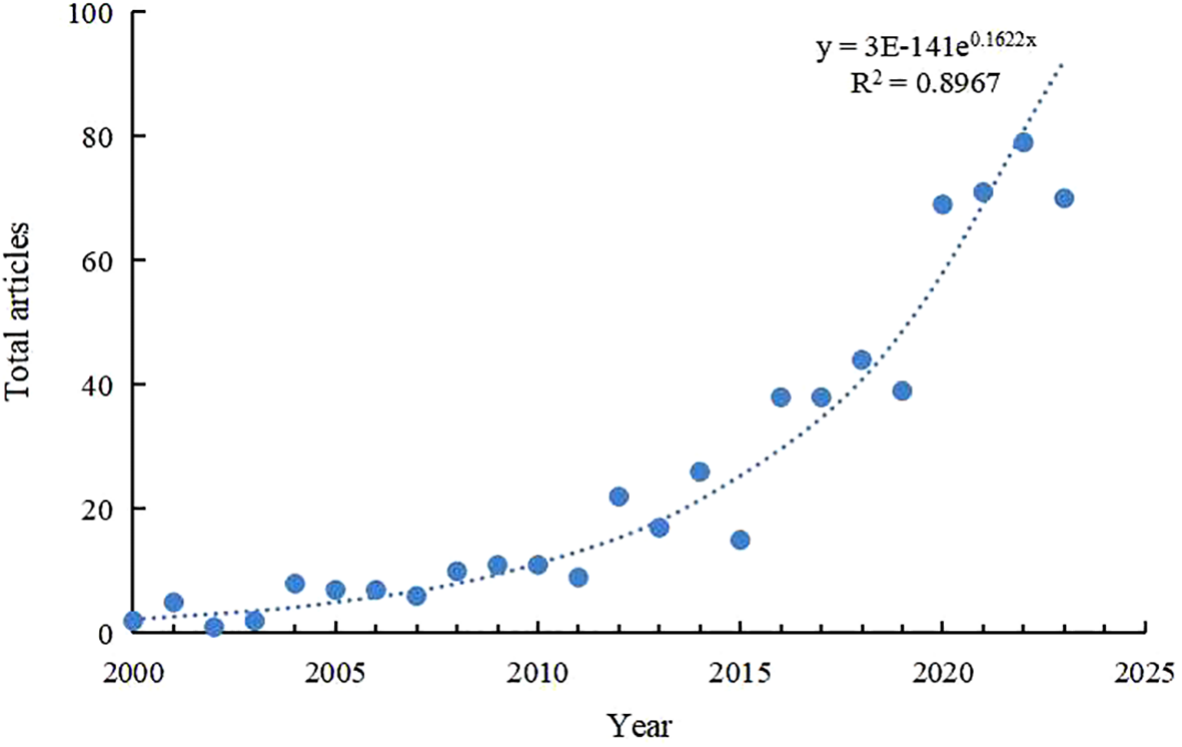
Fitting curve for annual publications.

As described in this paper, a bibliometric analysis was conducted on research concerning soil organic carbon components reported from 2000 to 2023, revealing the trajectory of findings published in this field. Our analysis has allowed the following conclusions to be drawn:

1. An in-depth analysis of published manuscripts revealed that China significantly leads other countries in terms of the impact on the research field of soil organic carbon components. Notably, Chinese institutions, including the Chinese Academy of Sciences, the Northwest Agriculture and Forestry University, and the University of Chinese Academy of Sciences, dominate the list of top research institutions. The collaborations among these leading institutions also underscore China’s pivotal role in research in this field.

2. The rate of growth in the volume of publications highlights the trajectory of the impact of the field of ecological variation on research on ecosystem carbon stocks into three distinct phases: the nascent phase (2000–2007), the preliminary exploration phase (2011–2019), and the phase of accelerated development (2020 to the present). **Figure 8** shows that R^2^ fitted to the annual publication curve reached 0.89, supporting the sustained international interest in organic carbon components. Indeed, the field of soil organic carbon components is now poised for rapid advancement, with the advent of innovative theories, methodologies, and technological breakthroughs. In line with this, researchers in adjacent disciplines should persistently engage with this burgeoning topic.

3. Current research hotspots within the field of soil organic carbon components are focused predominantly on “the response of soil organic carbon components to vegetation restoration”, “the impact of straw return on soil organic carbon components”, and “soil organic carbon in the Loess Plateau and karst landscapes”. The concentration on these themes is intended to elucidate the dynamics of changes in soil organic carbon across various countries via collaborative research endeavors, demonstrating a significant level of interdisciplinary synthesis.

In view of current hotspots and gaps in research, future research within “soil organic carbon components” should focus on the following three points: 1) future research should aim to extensively explore the complex components that constitute soil organic carbon. There is currently a lack of a universally accepted precise standard for categorizing organic carbon components, which has resulted in significant overlap and ambiguity in the delineation of their respective roles within existing classifications. Researchers globally, with expertise in pertinent disciplines, are well positioned to further elucidate the composition and origins of soil organic carbon. This exploration encompasses investigations of diverse types of organic matter such as microbial tissues, humins and plant residues, and their distribution patterns and interrelationships within the soil matrix. Moreover, advancements in sophisticated technologies, including nanotechnology, promise to enhance the precision and sensitivity with which organic carbon can be detected. It is imperative to explore the potential applications of nanomaterials in facilitating the transformation and enhancement of organic carbon stocks. Moreover, the coupling of spectral analysis with high-throughput sequencing of microbial macro-genomes could provide detailed insights into the microbial dynamics that shapes soil organic carbon components. This approach would provide a clearer understanding of the overlapping relationships among various organic carbon components and facilitate the establishment of a quantitative discrimination model. Concurrently, there is an urgent need for innovations in techniques for monitoring carbon in the soil. These can include the use of remote sensing technology coupled with stereoscopic soil carbon measurements. Additionally, integrating these methodologies to develop innovative approaches such as data mining and machine learning should improve the speed and accuracy with which soil organic carbon components can be assessed, enabling large-scale monitoring efforts.

2) Since the 1980s, global climate change has attracted increasing attention, leading to in-depth investigations into the potential impact of soil organic carbon pools on greenhouse gas emissions and climate change. With the deepening understanding of the global carbon cycle and climate systems, research on soil carbon pools has similarly intensified, shifting from a singular focus on overall soil organic carbon pools to a more granular examination of individual soil organic carbon components. However, previous studies on carbon pools typically focused on single-factor effects. Future research should aim to improve the parameterization of soil carbon inputs, transformations, and losses while integrating multiple factors such as climate, land-use change, and land management practices. This will enable a more comprehensive analysis of agricultural soil carbon cycling and further optimization of soil carbon cycle models, leading to better predictions of soil organic carbon dynamics in the context of global climate change.

3) In conclusion, future research on soil organic carbon components should be predicated on a deeper understanding of their composition and properties while also strengthening international cooperation and exchange. Through experiments and long-term monitoring, combined with longitudinal experimental data and high-precision measurements of soil organic carbon components, dynamic models of these components should be refined and validated. Additionally, research findings on the impact of soil organic carbon components on carbon stability and cycling should be integrated, along with the application of modern advanced technologies. In this context, it is imperative to investigate the response mechanisms of soil organic carbon components on carbon sequestration and release across various terrestrial ecosystems on a global scale. It is also crucial to explore the interactions between soil organic carbon and soil biology, physical structure, and chemical properties, and their effects on plant growth and soil health. Establishing a global network for research on soil organic carbon components and enhancing global carbon sequestration and carbon management strategies will further propel the development of sustainable soil management, carbon cycle research, and strategies for adapting to global climate change.
